# ATHENA: Identifying interactions between different levels of genomic data associated with cancer clinical outcomes using grammatical evolution neural network

**DOI:** 10.1186/1756-0381-6-23

**Published:** 2013-12-20

**Authors:** Dokyoon Kim, Ruowang Li, Scott M Dudek, Marylyn D Ritchie

**Affiliations:** 1 Center for Systems Genomics, Department of Biochemistry and Molecular Biology, Pennsylvania State University, University Park PA, USA

**Keywords:** Integrative analysis, Multi-omics data, Grammatical evolution neural network, Ovarian cancer

## Abstract

**Background:**

Gene expression profiles have been broadly used in cancer research as a diagnostic or prognostic signature for the clinical outcome prediction such as stage, grade, metastatic status, recurrence, and patient survival, as well as to potentially improve patient management. However, emerging evidence shows that gene expression-based prediction varies between independent data sets. One possible explanation of this effect is that previous studies were focused on identifying genes with large main effects associated with clinical outcomes. Thus, non-linear interactions without large individual main effects would be missed. The other possible explanation is that gene expression as a single level of genomic data is insufficient to explain the clinical outcomes of interest since cancer can be dysregulated by multiple alterations through genome, epigenome, transcriptome, and proteome levels. In order to overcome the variability of diagnostic or prognostic predictors from gene expression alone and to increase its predictive power, we need to integrate multi-levels of genomic data and identify interactions between them associated with clinical outcomes.

**Results:**

Here, we proposed an integrative framework for identifying interactions within/between multi-levels of genomic data associated with cancer clinical outcomes using the Grammatical Evolution Neural Networks (GENN). In order to demonstrate the validity of the proposed framework, ovarian cancer data from TCGA was used as a pilot task. We found not only interactions within a single genomic level but also interactions between multi-levels of genomic data associated with survival in ovarian cancer. Notably, the integration model from different levels of genomic data achieved 72.89% balanced accuracy and outperformed the top models with any single level of genomic data.

**Conclusions:**

Understanding the underlying tumorigenesis and progression in ovarian cancer through the global view of interactions within/between different levels of genomic data is expected to provide guidance for improved prognostic biomarkers and individualized therapies.

## Background

Cancer, a complex disease of somatic mutations and regulation abnormalities, causes substantial gene expression changes in its tumor cell [1]. Expression of oncogenes or tumor suppressor genes promotes the malignant phenotype of cancer cells or inhibits cell division, development, or survival of cancer cell [1]. Gene expression profiles have been broadly used in cancer research as a diagnostic or prognostic signature for the clinical outcome prediction such as stage, grade, metastatic status, recurrence, and patient survival, in addition to potentially improving patient management [2-6]. In terms of translational bioinformatics, accurate outcome prediction based on the molecular signature can be used clinically to choose the best of several available therapies for a cancer patient. For example, a high risk patient may be advised to select a more radical therapy.

However, emerging evidence shows that gene expression-based prediction varies between independent data sets and little is known about the accuracy of gene expression-based prediction model with distinguished pathologic and clinical predictors [7,8]. One possible explanation of this effect is that previous studies were focused on identifying genes with large main effects associated with clinical outcomes. Thus, non-linear interactions, which can be a candidate of synthetic lethal interactions, without large main effects would be missed [9]. The other possible explanation is that gene expression as a single level of genomic data is insufficient to elucidate the clinical outcome since cancer can be dysregulated by multiple alterations through genome, epigenome, transcriptome, and proteome levels [10].

Recently, the emerging data generation of genomic data has provided unprecedented opportunities to investigate the global view of complex mechanisms between multi-layers of genomic data. The Cancer Genome Atlas (TCGA) is a large-scale collaborative initiative to improve the understanding of cancer using meta-dimensional genomic data. The TCGA research network recently published many notable papers on several cancers concerning an interim analysis of DNA sequencing, copy number, DNA methylation, miRNA, and gene expression data [11-15]. The International Cancer Genome Consortium (ICGC) is another multidisciplinary collaborative initiative to characterize a comprehensive description of genomic, transcriptomic and epigenomic abnormalities in 50 different cancer types [16]. While the TCGA and ICGC open many opportunities to deepen the knowledge of the molecular basis of cancer [16-19], it is particularly important to integrate different levels of genomic data at hand for providing an enhanced global view on interplays between them.

In order to overcome the variability of diagnostic or prognostic predictors from gene expression data and to increase its predictive power, we need to integrate multi-levels of genomic data and identify interactions between them associated with clinical outcomes. Interactions within a single genomic level such as gene-gene interaction, miRNA-miRNA interaction, or protein-protein interaction have been known to be associated with cancer susceptibility, progression, and treatment [9,20-23]. In addition, interactions between multi-levels of genomic data such as miRNA-target gene interaction, copy number-gene interaction, or methylation-gene interaction are also associated with cell development, stress response, apoptosis, proliferation, and tumorigenesis [24-26]. However, to the best of our knowledge, there is no systematic approach to identify interactions within/between different levels of genomic data for cancer clinical outcome prediction.

In this study, we proposed an integrative framework for identifying not only interactions within a single genomic level but also interactions between multi-levels of genomic data associated with cancer clinical outcomes using the grammatical evolution neural networks. In order to highlight the validity of the proposed framework, ovarian cancer data from TCGA was used as a pilot task. Serous cystadenocarcinoma is the most prevalent form of ovarian cancer, and is the 5^th^ leading cause of cancer mortality in women in the United States [27]. Understanding the underlying biology and molecular pathogenesis in ovarian cancer survival through the global view of interactions between different levels of genomic data is expected to provide guidance for improved prognostic biomarkers and individualized therapies.

## Methods

### Data

Normalized datasets in ovarian cancer were retrieved from the Cancer Genome Atlas (TCGA) data portal (http://tcga-data.nci.nih.gov/) (Table 1). DNA methylation, gene expression, and miRNA expression data contain 27,578 CpG loci, 12,042 genes, and 799 miRNAs, respectively. Copy number alteration (CNA) data was obtained from cBio Cancer Genomics Portal in order to use the results of altered regions of amplification or deletion across sets of patients from GISTIC algorithm [28]. CNA data contains 54 significant cytoband regions. A binary classification of short-term and long-term survival was set as a pilot task. In the classification of *short-term or long-term survival*, ‘short-term’ represents the patients who survived less than 3 years, whereas ‘long-term’ indicates patients who survived longer than 3 years [29]. A total of 258 patients’ records were available across the CNA, methylation, miRNA, and gene expression data sets (*N* = 258) with survival information, in which 110 were short-term survival and 148 were long-term survival.

**Table 1:** Data description

**Data type**	**Platform**	**# Features**
CNA	Agilent SurePrint G3 human CGH microarray kit 1x1M	54 cytobands
Methylation	Infinium human methylation27 BeadChip	27,578 CpG loci
miRNA	Agilent human miRNA microarray Rel2.0	799 miRNAs
Gene expression	Affymetrix HT human genome U133 array plate set	12,042 genes

### Analysis Tool for Heritable and Environmental Network Associations (ATHENA)

We have developed ATHENA, a multi-functional software package, designed to perform the three main functions essential to determine the meta-dimensional models of complex disease: (1) performing feature/variable selections from categorical or continuous independent variables; (2) modelling main and interaction effects that explain or predict categorical or continuous clinical outcomes; (3) interpreting the significant models for use in further translational bioinformatics [30-32]. ATHENA contains filtering components, modelling components, and an evolutionary computing approach based on a machine technique to generate complex models. The current version of ATHENA has two different computational evolution modelling methods, Grammatical Evolution Symbolic Regression (GESR) and Grammatical Evolution Neural Networks (GENN).

**Figure 1:**
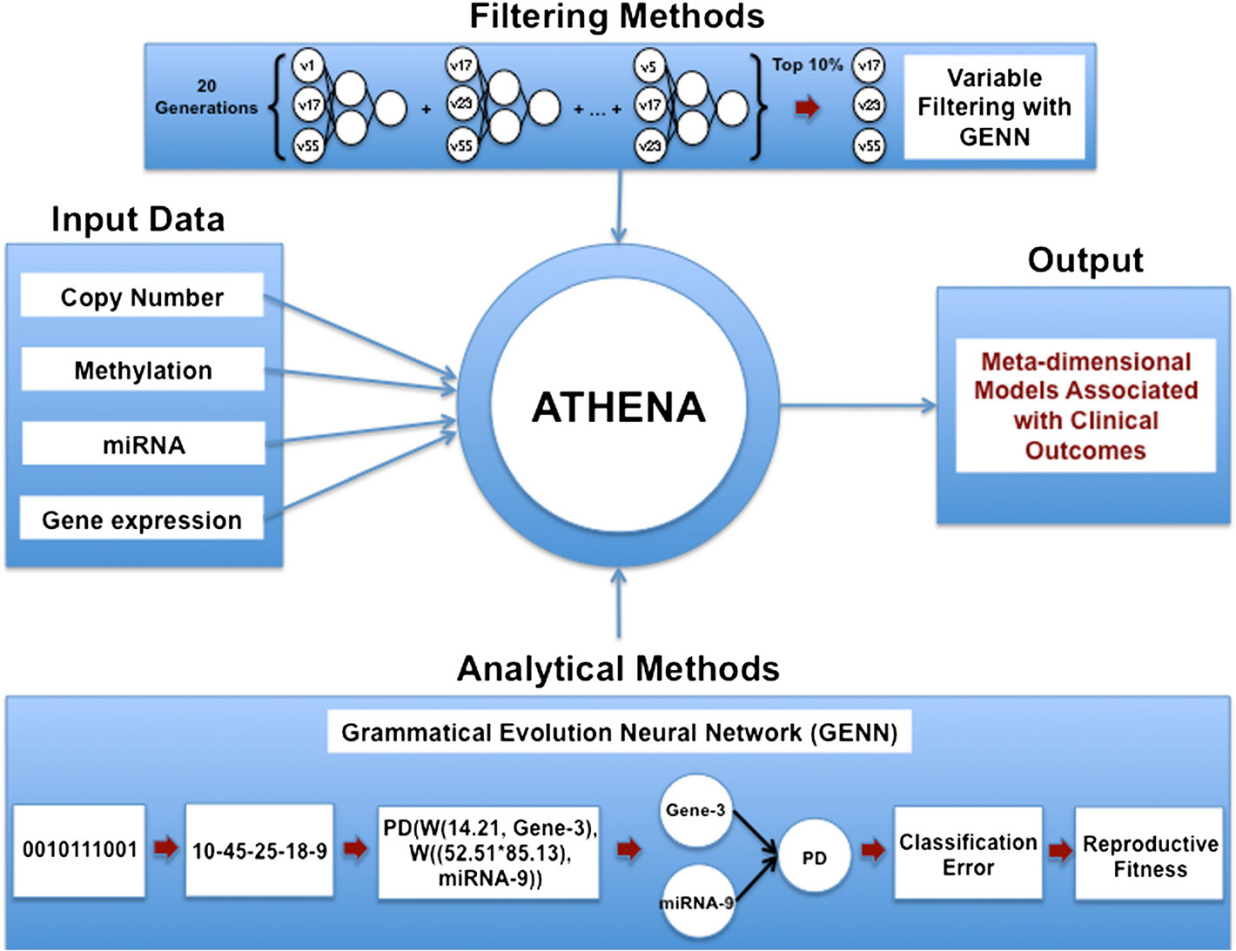
**Schematic overview of ATHENA.** ATHENA contains filtering and modelling components and different levels of genomic data can be the input for the meta-dimensional models associated with clinical outcomes of interest.

We have extended ATHENA to address the issue of integrating data from multiple “-omics” dimensions to identify models that explain the multi-layered architecture of complex traits. Figure 1 shows a schematic of the ATHENA methodology for the current task. In particular, multi-omics data such as CNA, methylation, miRNA, and gene expression data can be inputs for ATHENA in order to determine the meta-dimensional models of complex disease. For this analysis, we used GENN as the filtering and modelling component.

### Grammatical Evolution Neural Networks (GENN)

In order to identify non-linear interactions between genomic features with small/large main effects, various computational methods have been introduced such as the multi-factor dimensionality reduction (MDR) [33,34]. However, MDR performs an exhaustive analysis of every possible combination of interacting loci to generate multi-locus predictor models. The search spaces of all n-wise interacting features will increase exponentially when integrating with different levels of genomic data. Thus, stochastic methods employing evolutionary computing approaches have been developed and demonstrated to utilize the full dimensionality of the data without exhaustively searching all possible combinations of variables that influence complex traits [31,35,36].

Artificial Neural Network (ANN) is a flexible and robust machine learning technique inspired by the basic function and structure of neurons to solve complex problems. ANN is a good candidate for identifying interactions that influence variance in an outcome of interest since it is able to model complex and non-linear relationships between variables. However, the conventional approach for applying ANN to a classification problem is only to fit the network weights using a gradient descent optimization method such as backpropagation when given input variables and network architecture, which are not known a priori. In order to optimize the input variables, weights, and network structures simultaneously, evolutionary computing approaches have been proposed [31,36]. Genetic programming, a specialization of genetic algorithms, is an evolutionary algorithm-based methodology that uses concepts of survival of the fittest for evolving a fit solution from an original population of random solutions [37]. In particular, grammatical evolution is a more flexible alternative version of genetic programming since the binary string as a heritable material can be translated into a functional solution, or computer program, via grammar rules (Figure 1) [36]. The details of the grammar rules were described in a previous study [36]. The GENN algorithm is briefly described as follows:

(1)  The original dataset is divided into 5 equal groups for 5-fold cross-validation (4/5 for training and 1/5 for testing dataset).

(2)  Training begins by generating a random population of binary strings initialized to be functional ANNs. The total population is divided into demes as sub-populations across a user-defined number of CPUs for parallelization.

(3)  The ANNs in the population are evaluated using the training data and the fitness (balanced classification accuracy) for each model is recorded. A new population is generated as the solutions with the highest fitness are selected for crossover and reproduction.

(4)  Step 3 is repeated for a pre-defined number of generations. Migration of best solutions occurs between demes every n-number of generations, as specified by user.

(5)  The overall best solution across generations is tested using the remaining 1/5 test dataset and fitness is recorded.

(6) Steps 2-5 are repeated four more times, each time using a different 4/5 of the data for training and 1/5 for testing. The best model is defined as the model identified the most over all five cross-validations.

Table 2 shows the GENN parameters for the analysis.

**Table 2:** GENN parameter settings

**Parameter**	**Value**
Number of demes (CPUs)	50
Population size/deme	5,000
Number of generations	300
Number of migrations	15
Probability of crossover	0.9
Probability of mutation	0.01
Fitness function	Balanced accuracy

### Experiment setup

**Figure 2:**
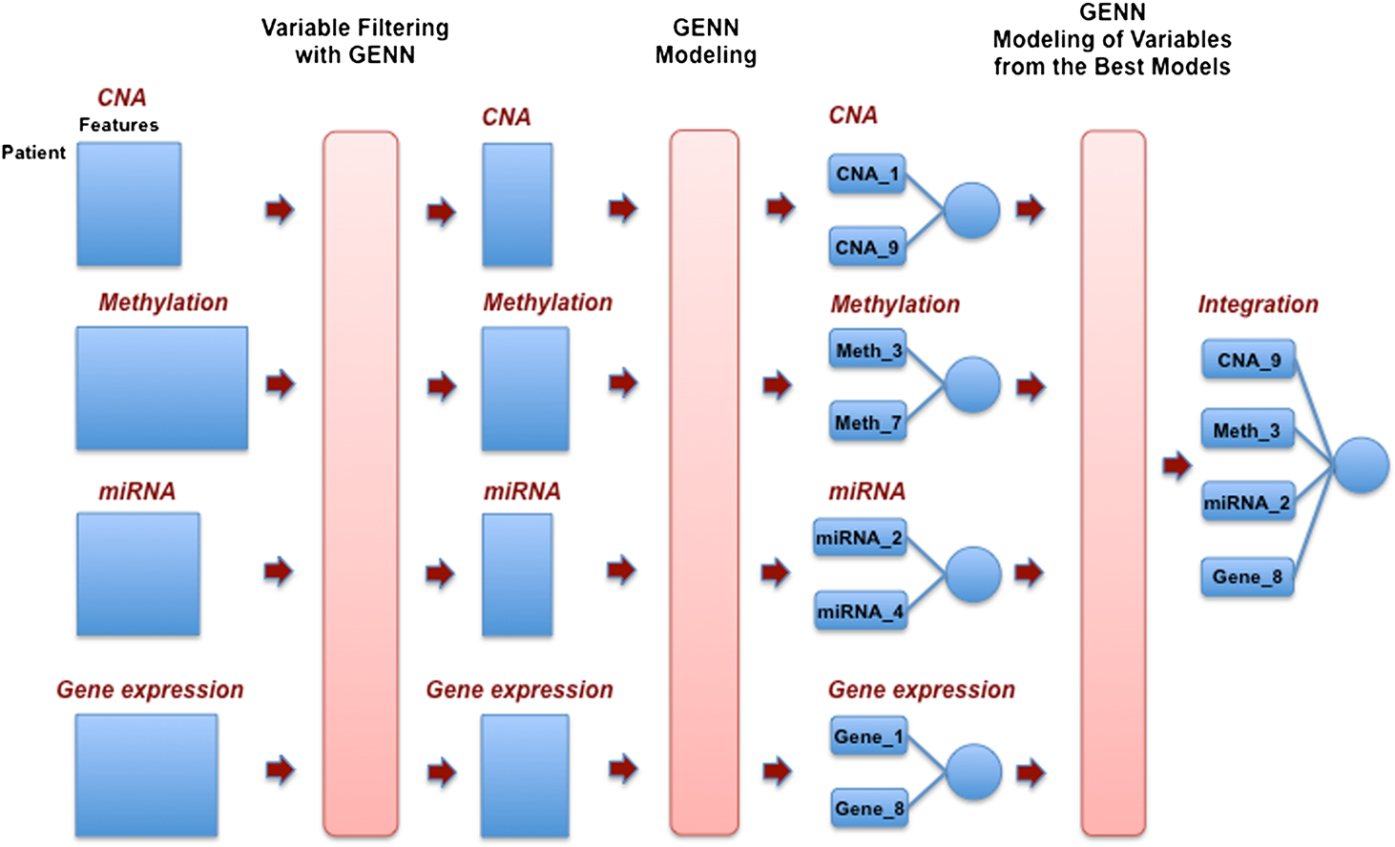
**Schematic overview of the pipeline for the analysis.** (1) Filtering the noise variables from each genomic data (2) GENN modeling (3) GENN modeling of variables from the best models of each genomic dataset.

Figure 2 shows the overview of the pipeline, which consists of a filtering step and a modelling step. We applied the filtering step to reduce the noise in the dataset since GENN has been shown to outperform other methods when the noise is reduced [38]. For the filtering step, GENN was run with 20 generations and 5,000 population size to generate many intermediate models for each genomic data (Figure 1). Then, the frequencies of each variable were calculated from all the intermediate models from each cross validation, and the features were ranked based on the frequency. We set three different thresholds, top 10%, top 30%, and top 50% of total variables based on their frequencies, in order to filter out the noise in the dataset. After the filtering step, we analysed the filtered dataset to generate the best predictive model using GENN. Finally, we integrated the best model from different levels of genomic data to determine the meta-dimensional model associated with survival. The balanced accuracy, which avoids inflated performance estimates on imbalanced datasets, was used in GENN as a fitness function.

## Results and discussion

### Filtering features

**Figure 3:**
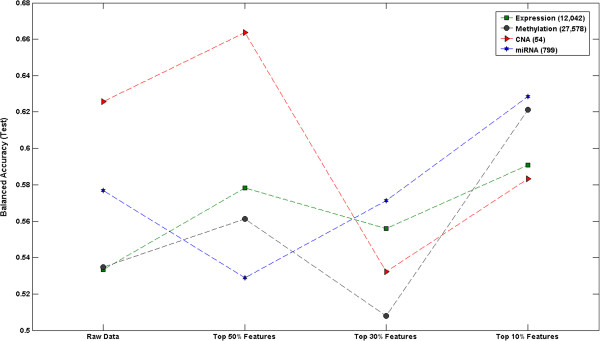
**The results of filtering step with different threshold for each genomic dataset.** Y axis refers to the balanced accuracy in the test dataset and x axis represents the different threshold for filtering variables.

In order to have a comparable threshold for each genomic dataset, we set different cut-offs of total variables from the intermediate models, top 10%, top 30%, and top 50%, respectively. GENN with 1,000 population size per deme and 10 demes was employed for each genomic dataset with different threshold in order to determine the best filtered dataset prior to modelling. Figure 3 shows the results of filtering steps with different cut-offs. Methylation, miRNA, and gene expression data showed the great improvement with top 10% threshold compared to the model with raw dataset, whereas CNA data with top 50% threshold showed the best performance among different cut-offs. Since the original number of variables in CNA data is relatively small than other dataset, top 10% or top 30% threshold were likely to filter out not only noise but true signals. Top 10% cut-off for methylation, miRNA, and gene expression dataset and top 50% cut-off for CNA dataset were set for the further study.

### GENN modelling for single level of genomic data

**Figure 4:**
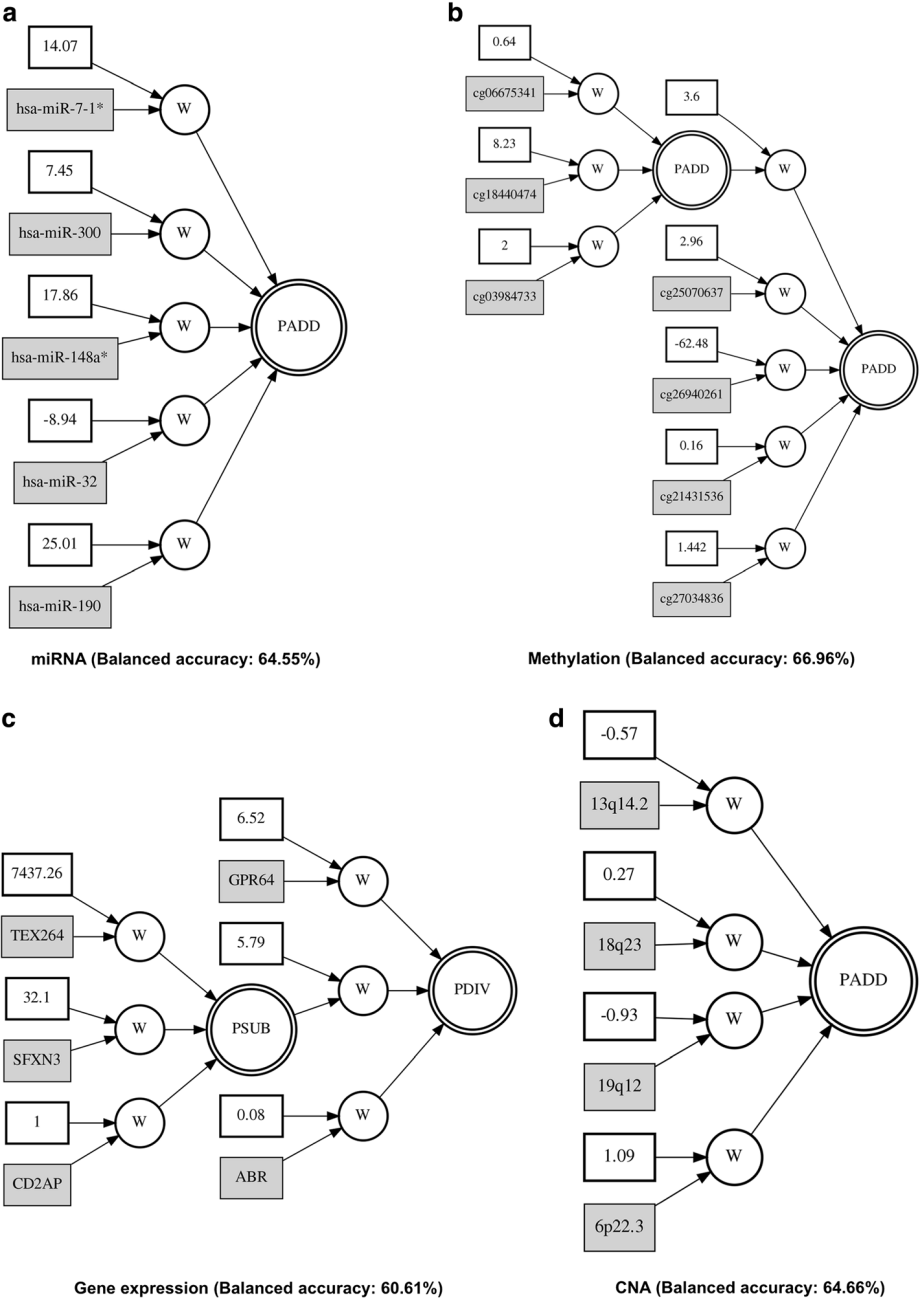
**Best GENN models from each genomic dataset.** PADD, PSUB, and PDIV are an addition, subtraction, and division activation node, respectively. Constants in the white boxes are weights. Genomic features such as miRNA, CpG loci, gene, and CNA cytoband, are shown in the gray boxes. **(a)** miRNA **(b)** Methylation **(c)** Gene expression **(d)** CNA dataset.

The filtered individual levels of genomic data, copy number alteration loci, CpG loci, miRNAs, and gene expression, were analysed separately by GENN with parameters described in Table 2. GENN is an evolutionary computing approach to evolve neural networks for predicting the clinical outcomes of interest by optimizing the input variables, weights, and network structure simultaneously. Thus, the final solution of GENN is the neural network with optimized input variables, weights, and network structure. Figure 4 shows the results of best ANN models from each genomic dataset: miRNA, methylation, gene expression, and CNA data, respectively. The best models from each genomic dataset showed different network structures, indicating complex interactions between genomic features within a single genomic level. The balanced accuracy values from the testing cross-validation set for each of the models with miRNA, methylation, gene expression, and CNA were 64.55%, 66.96%, 60.61%, and 64.66% of balanced accuracy, respectively.

### Integration with different levels of genomic data

**Figure 5:**
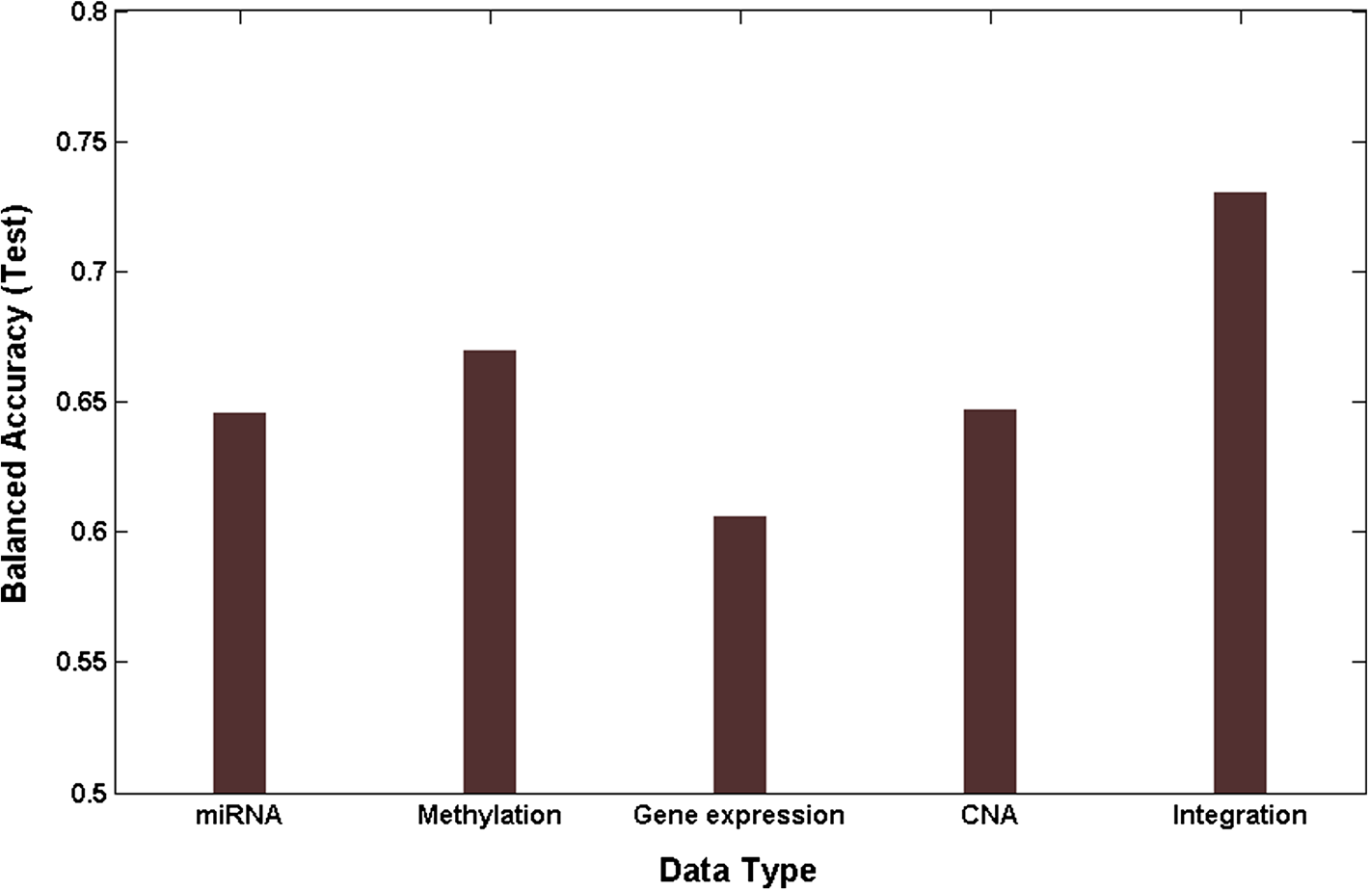
**Performance comparison between integration model and the model with single level of genomic data.** Integration model was conducted by combining variables from miRNA, methylation, gene expression, and CNA data.

**Figure 6:**
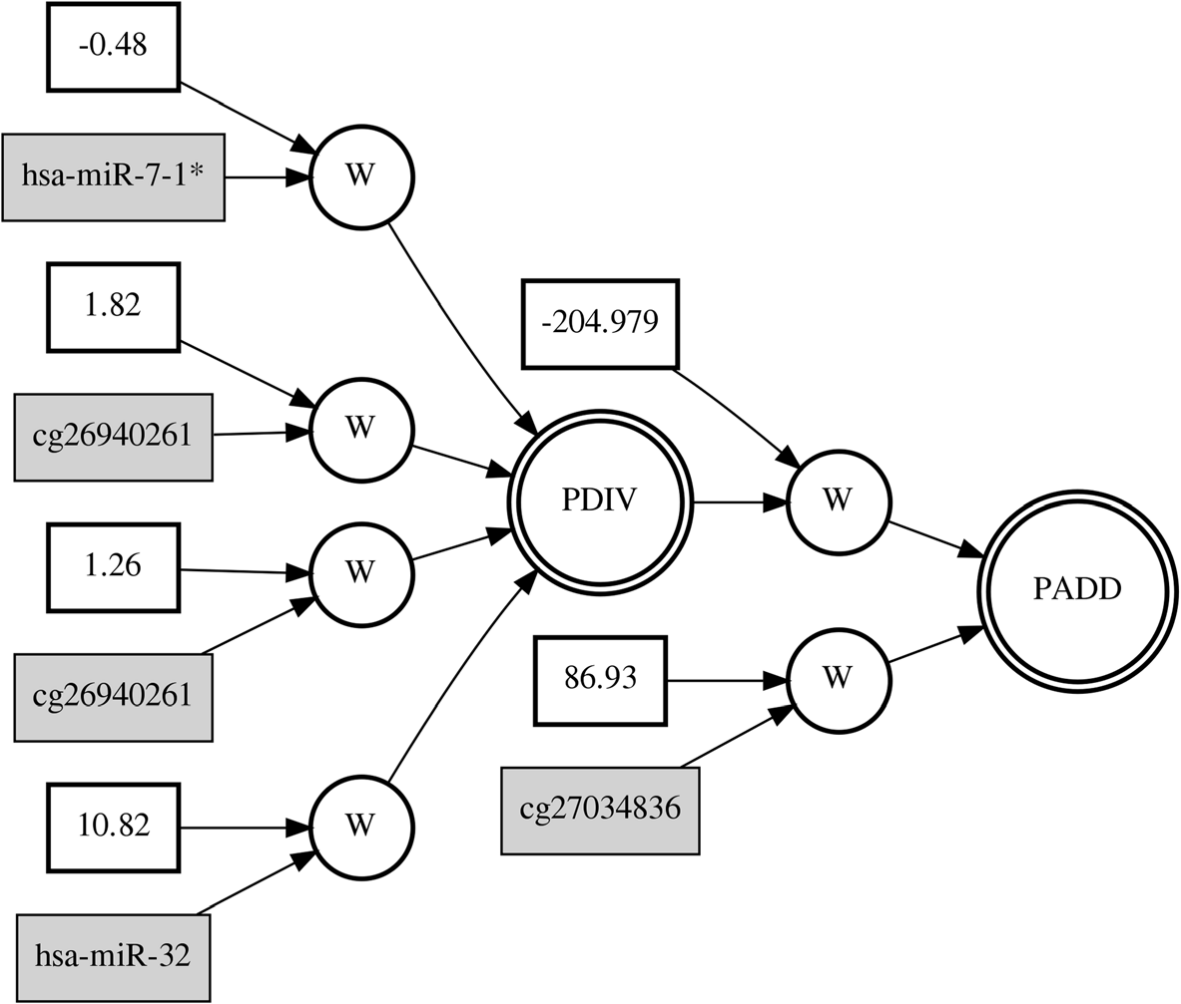
Best GENN model of variables from different levels of genomic data.

We integrated miRNA, methylation, gene expression, and CNA data in order to identify interactions between different levels of genomic data associated with survival in ovarian cancer. The final multi-dimensional model was conducted from GENN with variables from the best models of each individual genomic dataset. The predictive power of integration showed the improvement compared to the model with single level of genomic data (Figure 5). The best multi-dimensional model of all variables from omics dimension was obtained with a balanced accuracy of 72.89% (Figure 6). The selected features in the final model are hsa-miR-32, hsa-miR-7-1*, cg26940261, and cg27034836 with variable consistency among 5 cross-validations, 2/5, 4/5, 4/5, and 3/5, respectively. Even though two of models among 5 cross-validations contain miRNA, methylation, gene, and CNA features, the predictive power was not as good as to the best model with 2 miRNAs and 2 CpG loci. In order to assess the significance in performance between the models of single level of genomic data and model of integration, the Wilcoxon singed-rank test was used (Table 3) [39]. All balanced accuracy values from 5-fold cross validation were used for the comparison between models.

**Table 3:** Significance test of the performances between the integration model and the model with single level of genomic data

**Comparison between models**	**p-value**
Integration vs. miRNA	0.0873
Integration vs. methylation	0.0476
Integration vs. gene expression	0.0159
Integration vs. CNA	0.1349

### Biological implication

Five miRNAs, hsa-miR-7-1*, hsa-miR-300, hsa-miR-148a*, hsa-miR-32, and hsa-miR-190, were found in the GENN models associated with survival in ovarian cancer. In general, the aberrant miRNA expression provides substantial consequences for the progression of tumorigenesis [40]. The miRNAs, hsa-miR-7-1*, hsa-miR-148a*, and hsa-miR-32, from the model were found to be a prognostic indicator in several cancers [19,41,42]. Synergistic regulations between miRNAs through either targeting same genes or co-operating of targeted genes are thought to be important to understand the mechanisms of complex post-transcriptional regulations since complex diseases such as cancer are affected by several miRNAs rather than a single miRNA [22]. In addition, we found possible interactions between genomic loci, 13q14.2, 18q23, 19q12, and 6p22.3, which are associated with survival in ovarian cancer. Identifying interactions between altered genomic loci is a prerequisite to detect any common pathways that may be deregulated through the alterations in gene copy number, suggesting co-operative or complementation effect related to the tumorigenesis [43,44].

Even though models from miRNA and CNA data showed additive effects, the models from methylation and gene expression data showed complex and non-linear interactions between genomic features associated with survival. In terms of epigenetic regulation, DNA methylation can serve to regulate expression of oncogene or tumor suppressor gene in cancer. Recently, ‘*epigenetic epistatic interactions*’ have been regarded to place important constrains on the evolution of gene expression that affects disease phenotype [45]. The non-linear interactions of methylation of genes, PRMT3, CHN1, HDHD3, SDC2, C12orf75, RXFP2, and GLB1, might contribute on the survival in ovarian cancer rather than the single methylation of a specific gene. Several genes including SDC2 as a methylation cluster are involved in activation of TGF-beta pathway in ovarian cancer [46]. A role for the insulin-relaxin family of peptides including INSL3 and its receptor RXFP2 in several cancers has been reported [47,48]. Similarly, complex interactions of genes, TEX264, SFXN3, CD2AP, GPR64, and ABR, might act on crucial role in molecular pathogenesis, progression, and prognosis of ovarian cancer through the expression.

**Figure 7:**
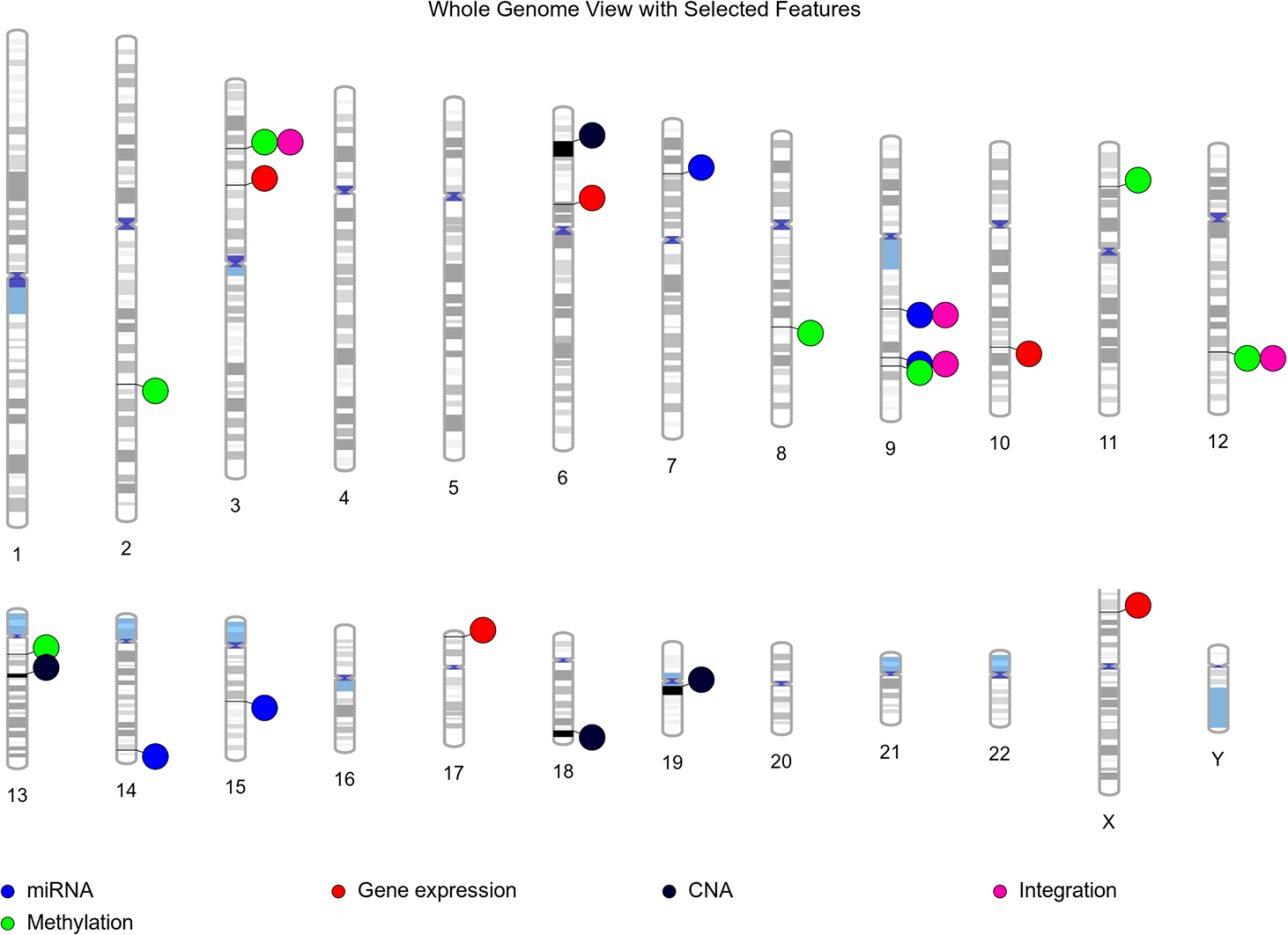
Whole genomic view of selected features in the GENN models.

In the final multi-dimensional model, 2 miRNAs, hsa-miR-32 and hsa-miR-7-1*, and 2 methylation probes, cg26940261 and cg27034836, were selected. Notably, cg27034836 is at the promoter CpG island of GLB1 gene and hsa-miR-32 targets GLB1 gene, which was obtained from MicroCosm database [49]. It suggests that there might be possible synergistic mechanism between methylation and miRNA regulation for the expression of GLB1 gene, encoding beta-galactosidase-1 that cleaves the terminal beta-galactose from ganglioside substrates and other glycoconjugates. Senescence-associated β-galactosidase activity in cancer cells induced to enter senescence requires expression of the GLB1 gene [50]. In order to provide the whole genome view, all the features from the best GENN model were plotted using Phenogram visualization software (Figure 7).

## Conclusions

In this study, we addressed the issue of integrating meta-dimensional genomic data and identifying complex interactions in order to overcome the variability of diagnostic or prognostic predictors from any single level of genomic data and to increase its predictive power. Here, we proposed an integrative framework for identifying interactions within/between multi-levels of genomic data associated with cancer clinical outcomes based on the grammatical evolution neural networks. GENN, an efficient evolutionary computing approach, has been shown to be powerful in genetic association studies and meta-dimensional analysis of phenotypes of interest and has been proven superior compared to other methods in term of prediction accuracy [31,32,36,38].

In order to demonstrate the utility of the proposed framework, ovarian cancer data from TCGA was used as a pilot task. We found not only interactions within a single genomic level but also interactions between multi-levels of genomic data associated with survival in ovarian cancer. Notably, the meta-dimensional model outperformed the model with single level of genomic data only. Taken together these results suggest that meta-dimensional model will lead us to an enhanced global view on interplays since different levels of genomic data might affect the cancer phenotype through either partly independent or partly complementary fashion. Understanding the underlying tumorigenesis and progression in ovarian cancer through the global view on interactions within/between different levels of genomic data is expected to provide guidance for improved prognostic biomarkers and individualized therapies. For instance, these models could be a candidate of synthetic lethal interaction, which is a new way in the context of anticancer therapy [9].

One of the limitations in the current study is that the final meta-dimensional model was obtained using variables from the best model of each genomic dataset. Thus, there will be a possibility to miss the interactions between different levels of genomic data, which were not selected in the best model because of small effect within a single genomic level. Another limitation of our analysis is the modeling techniques do not specifically identify conditional relationships, which are likely to be ubiquitous in meta-dimensional data. For example, if miRNA affect expression level of its target gene, which, in turn, affected the phenotype, methods such as GENN are more likely to identify either miRNA or gene expression, but not both. Bayesian networks could model these types of relationships in a more informative manner. Future improvement to ATHENA will include incorporating Bayesian networks to allow for the generation of more interpretable meta-dimensional models. Moreover, even though the current study was set for the classification problem between short-term and long-term survival, GENN is also able to predict continuous clinical outcomes. However, continuous survival data could not be directly used in GENN due to the context of censored data. In addition, in the current implementation of GENN and in evolutionary algorithm in general, the norm is to select the best model in the final solution because it has higher accuracy than all of the other models. However, there might be multiple different good models and selection based on accuracy alone has its limitations. To overcome this limitation, Pareto optimization can be incorporated in the next iteration of GENN. Pareto optimization is a multi-objective optimization method that aims to maximize or minimize multiple objectives. In our case, through minimizing the model size and the error, it will produce an array of equally good models that are not dominated by other models. Pareto optimization will allow us to find multiple interactions in cancer. We leave these investigations about the alternative way of integration, capturing the conditional relationship, predicting continuous survival data, and Pareto optimization as our future works. Another interesting direction for further works would be the integration with biological knowledge as a knowledge-driven approach.

Even though the current study is limited to the prediction of short-term/long-term survival in ovarian cancer as a base task, the proposed framework can be applied to other clinical outcomes such as stage, recurrence, metastasis, grade, *etc*. Furthermore, it can be applied to other cancer types in order to identify the cancer-specific or common interactions among cancer types. With abundance in multi-omics data and clinical data from TCGA or ICGC, our proposed framework will be valuable for explaining novel tumorigenesis, eventually leading to more effective screening strategies and therapeutic targets in many types of cancer. ATHENA can be downloaded from http://ritchielab.psu.edu/ritchielab/software/.

## Competing interests

All authors declared that there is no conflict of interest in this research.

## Authors’ contributions

DK and MDR designed and developed the study and wrote the manuscript. DK, RL and SMD provided the experimental results and interpreted the results. MDR provided intellectual guidance and mentorship and wrote the manuscript. All authors read and approved the final manuscript.
